# Association Between Lifetime Affective Symptoms and Premature Mortality

**DOI:** 10.1001/jamapsychiatry.2020.0316

**Published:** 2020-04-08

**Authors:** Gemma Archer, Diana Kuh, Matthew Hotopf, Mai Stafford, Marcus Richards

**Affiliations:** 1 MRC Unit for Lifelong Health and Ageing at UCL, University College London, London, United Kingdom; 2 Department of Psychological Medicine, Institute of Psychiatry, Psychology & Neuroscience, King’s College London, London, United Kingdom; 3 South London and Maudsley National Health Service Foundation Trust, London, United Kingdom

## Abstract

**Importance:**

Associations between affective symptoms and mortality have been evaluated, but studies have not examined timing or cumulative exposure to affective symptoms over the life course.

**Objectives:**

To examine how lifetime accumulation and timing of affective symptoms are associated with mortality and identify potential explanatory factors.

**Design, Setting, and Participants:**

Data were obtained from the MRC National Survey of Health and Development (1946 British birth cohort), a socially stratified, population-based sample originally consisting of 5362 singleton births in England, Wales, and Scotland during March 1946. The cohort has been followed up 24 times, most recently in 2014-2015. Eligible participants included those flagged for mortality with affective symptom data available at a minimum of 3 time points (n = 3001). Data analysis was conducted from July 2016 to January 2019.

**Exposures:**

Affective symptoms were assessed at ages 13 to 15 years (teacher-rated questionnaire), 36 years (Present State Examination clinical semistructured interview), 43 years (Psychiatric Symptom Frequency questionnaire), and 53 years (General Health Questionnaire–28). Case-level affective symptoms were determined by those scoring in the top 16th percentile (ie, suggestive of a clinical diagnosis).

**Main Outcomes and Measures:**

Mortality data were obtained from the UK National Health Service Central Register from age 53 to 68 years.

**Results:**

Of 3001 study members (1509 [50.3%] female, 1492 [49.7%] male), 235 individuals (7.8%) died over a 15-year follow-up. After adjustment for sex, those who experienced case-level affective symptoms 1, 2, and 3 to 4 times had 76%, 87%, and 134% higher rates of premature mortality, respectively, compared with those who never experienced case-level symptoms. Case-level symptoms in adolescence only (ages 13-15 years) were associated with a 94% increased rate of mortality, which was unexplained after full adjustment for covariates (hazard ratio, 1.73; 95% CI, 1.10-2.72). Associations between participants with case-level symptoms multiple (2-4) times and mortality were predominately explained by adult health indicators and behaviors. For example, associations for those with case-level symptoms 3 to 4 times were most strongly attenuated by number of health conditions (32.1%), anxiolytic use (28.4%), lung function (24.6%), physical activity (23.9%), smoking (24.6%), antidepressant use (20.1%), diet (16.4%), pulse rate (12.7%), and adult social class (11.2%).

**Conclusions and Relevance:**

Lifetime accumulation of affective symptoms may be associated with an increased rate of mortality, with explanatory pathways dependent on the duration and timing of symptoms. Future research into causal pathways and potential points of intervention should consider affective symptom history.

## Key Points

**Question:**

How are affective symptoms over the life course associated with mortality?

**Findings:**

In a British birth cohort (n = 3001), affective symptoms were assessed between adolescence and age 53 years. Those who had case-level affective symptoms 1, 2, and 3 to 4 times had 76%, 87%, and 134% higher rates of premature mortality, respectively, by age 68 years compared with those who never experienced case-level symptoms; associations were largely explained by factors in adulthood, such as self-reported health conditions, smoking, and physical activity.

**Meaning:**

Future research into causal pathways and potential points of intervention for premature mortality should consider affective symptom history.

## Teaser

This cohort study examines mortality in individuals with varying levels of affective symptoms occurring over 15 years.

## Introduction

Associations between affective problems (anxiety and depression) and premature mortality have been demonstrated among psychiatric, medical, and community-based samples^1,2^ and across many causes of death^2,3^; however, most studies have relied on a single measure of affective symptoms in mid to late adulthood and a relatively short follow-up.^1,2,3^ Little is known about the long-term association between affective symptoms and mortality, particularly whether associations are determined more by accumulation or timing of symptoms. Of studies assessing affective symptoms over multiple time points, mortality^4,5^ has been estimated to increase with the frequency of case-level symptoms, but all of those studies were conducted in samples of older persons, with a maximum 7-year exposure window. Also, few studies have included a comprehensive range of potential major explanatory variables.^1^

We used data from the 1946 British birth cohort up to age 68 years, which has measures of affective symptoms spanning adolescence to late adulthood in addition to data on a wide range of potential explanatory factors. Our aims were to (1) examine the association between lifetime accumulation of affective symptoms and premature mortality, (2) investigate the role of symptom timing, and (3) examine whether associations were explained by a range of socioeconomic, behavioral, and physiological factors.

## Methods

The MRC National Survey of Health and Development (NSHD) is Britain’s oldest birth cohort, originally consisting of a socially stratified sample of 5362 singleton births in England, Wales, and Scotland during a single week in March 1946. The cohort has been followed up 24 times, most recently in 2013-2014 (age, 68-69 years),^6^ when its socioeconomic profile was broadly similar to a census reference population.^7^ Data analysis for the present study was conducted from July 2016 to January 2019. The most recent ethical approval was granted by the National Research Ethics Service Committee London Queen Square and by the Scotland A Research Ethics Committee. All study members gave written informed consent and did not receive financial reimbursement. This study followed the Strengthening the Reporting of Observational Studies in Epidemiology (STROBE) reporting guideline for cohort studies.

Mortality data were obtained from study records and linked with National Health Service Central Registry data. Follow-up time was from age 53 years to death or censored owing to emigration or October 31, 2014.

Affective symptoms were analyzed as a single construct because we found no evidence that mortality was associated with a particular type of symptom, such as anxiety vs depressive. Measures of affective symptoms were available at ages 13, 15, 36, 43, and 53 years. At ages 13 and 15 years, these symptoms were assessed by teachers using a forerunner of the Rutter B questionnaire.^8^ Factor scores were standardized at ages 13 and 15 years and then summed to create a single measure of adolescent affective symptoms.^9^

When study members were aged 36 years, affective symptoms were assessed using the short form of the Present State Examination,^10,11^ which is a validated, semistructured clinical interview designed to assess the frequency and severity of psychiatric symptoms in the preceding month.^12^ All items relating to anxiety and depressive disorders were extracted (Cronbach α = 0.83), and a factor score was created using confirmatory factor analysis.

Affective symptoms at age 43 years were elicited by trained interviewers using the Psychiatric Symptom Frequency scale, which is an 18-item questionnaire assessing the frequency and severity of anxiety and depressive symptoms in the preceding year.^13^ At age 53 years, affective symptoms were measured using the 28-item General Health Questionnaire, which is a validated, self-reported questionnaire assessing anxiety and depressive symptoms in the preceding 4 weeks.^14,15^

For each assessment, case-level affective symptoms were determined by those scoring in the top 16th percentile (ie, suggestive of a clinical diagnosis) based on the estimated prevalence of common mental disorders in the UK population.^16^ Affective case accumulation was obtained by adding the total number of times study members experienced a case at the ages tested (0, 1, 2, and 3-4 times).

Affective case histories were created to differentiate between potential proximal and distal effects among those who experienced case-level symptoms at a single time point. These categories included adolescent only (case-level symptoms at ages 13-15 years only) and late onset (case-level symptoms at age 53 years only). Other categories included never, chronic (case-level symptoms 3-4 times), and intermittent (case-level symptoms 3-4 times), and intermittent (case-level symptoms 1-2 times).

Potential explanatory factors were identified a priori as life course factors associated with affective disorders and mortality. Variables were obtained by postal questionnaire or nurse interview unless otherwise specified. Sociodemographic factors were sex, occupational social class at age 53 years (defined according to the Registrar General), and educational attainment by age 26 years.

Health indicators were assessed by trained nurses in participants aged 53 years and included systolic blood pressure, body mass index, lung function (forced expiratory volume in 1 second), and resting pulse rate. The number of self-reported health conditions, using the wording in the questionnaires, at ages 43 and 53 years (0, 1, 2, and ≥3 conditions) included ever having bronchitis, blood pressure problems, heart trouble, cancer, stroke, or diabetes, which were summed across ages.^7^

Health behaviors were smoking history (never, predominantly nonsmoker, predominantly smoker, and lifelong smoker^17^); average physical activity across ages 36, 43, and 53 years^18^ (inactive, moderately active, or most active); alcohol misuse at ages 43 or 53 years (using the CAGE [cutting down, annoyance by criticism, guilty feeling, and eye-openers] questionnaire^19^); and average dietary choice scores across ages 36, 43, and 53 years (obtained from 5-day diet diaries and calculated using the Eating Choices Index).^20,21^ The index scoring ranges from 4 to 20, with higher scores representing healthier choices, based on breakfast consumption, fruit portions, type of milk, and type of bread consumed.

Psychotropic medication included antidepressant and anxiolytic use at ages 31, 36, 43, or 53 years. Social networks included marital status at age 53 years and perceived social support at ages 43 and 53 years. Number of stressful life events in the preceding year were summed across ages 36, 43, and 53 years and categorized into 0 to 5, 6 to 9, and 10 or more events.^22^ Adverse childhood experiences were teacher-rated cleanliness of the child at age 11 years, used to indicate neglect; parental divorce or separation when the child was younger than 13 years; and parental abuse, retrospectively assessed when the participant was aged 43 years. Other childhood factors included social class (based on father’s social class at age 11 years), childhood sickness (based on absence from school at ages 6-12 years), and adolescent externalizing at ages 13 to 15 years.^9^ Presence of schizophrenia was ascertained by questionnaire, interview, and hospital and general practitioner contact data up to age 43 years.^23^

Participants included all those with complete affective symptom data at a minimum of 3 time points and who consented to National Health Service mortality flagging. Of the original birth cohort (N = 5362), 476 participants died when younger than age 53 years, 585 emigrated, 1266 refused participation or were unable to be traced, 12 had nonlinked mortality data, and 957 had missing affective symptom data at ages 13 to 15, 36, 43, or 53 years. Missing covariate data ranged from 0% to 8.6%, with the exception of childhood sickness absence (17.2%) and diet diary data (29.2%-43.1%). Multiple imputation using chained equations was used to impute missing data on covariates and those missing a single measure of affective symptoms,^24^ leaving 3001 study members in the analytical sample (509 [50.3%] female, 1492 [49.7%) male, 235 individuals (7.8%) died over a 15-year follow-up. eTable 1 in the Supplement provides the characteristics of observed and imputed samples.

### Statistical Analysis

Kaplan-Meier graphs were used to compare the survival probability of individuals with case-level affective symptoms 0, 1, 2, and 3 to 4 times over the follow-up period. The association between affective case accumulation and all-cause mortality rates were assessed using Cox proportional hazards models. First, the model was adjusted for sex. Second, each covariate was entered into the sex-adjusted model. Third, a final model adjusted for all covariates simultaneously. The extent to which each adjustment attenuated associations was calculated by 100 − [(aHR − 1)/(HR − 1) × 100], where aHR indicates adjusted hazard ratio.

The above analyses were repeated using the affective case history variable. Piecewise Cox regression examined the association between affective case history and mortality by follow-up time. Sex interactions were tested using joint Wald tests; there was no evidence that associations differed between men and women.

Sensitivity analyses were conducted by excluding schizophrenia cases and external causes of death (suicide, unintentional, or violence). With 2-tailed testing, findings were considered statistically significant at α = .05. All analyses were carried out in Stata, version 14 (StataCorp LLC).

## Results

Mean follow-up time was 14.4 years (range, 0.1-15.0 years), during which there were 235 deaths (102 men and 133 women). Of these, 20 were due to external causes (violence, unintentional, or suicide).

Table 1 reports the characteristics of the study sample by affective case-level symptom accumulation. With the exception of childhood social class, most covariates were associated with case accumulation (eg, antidepressant use: 4.5% for participants with no symptoms vs 37.8% for those with 4 case-level affective symptoms), and most by dose-response, with weaker associations observed for pulse rate, body mass index, alcohol misuse, diet, and adolescent externalizing.

**Table 1.** Descriptive Characteristics of 3001 Study Participants by Affective Case Accumulation Based on 15 Imputations

Exposure	No. of affective cases				
	0	1	2	3-4	*P* value^a^
Women, %	43.1	55.5	66.1	71.9	<.001
Lowest adult social class (V), %^b^	2.8	4.5	5.6	6.7	<.001
No educational qualifications, %	34.0	39.8	37.8	45.9	<.001
≥3 Health conditions, %^c^	5.5	8.3	12.6	17.4	<.001
Lung function, mean (SD), FEV_1_, L	2.81 (0.70)	2.63 (0.72)	2.44 (0.70)	2.36 (0.64)	<.001
Systolic blood pressure, mean (SD), mm Hg	137.4 (19.8)	135.2 (20.1)	134.5 (19.7)	130.6 (18.3)	.001
Pulse rate, mean (SD), bpm	68.0 (11.4)	68.5 (11.8)	68.7 (11.7)	69.4 (12.5)	.10
BMI, mean (SD)	27.4 (4.5)	27.5 (5.0)	27.7 (5.5)	28.1 (5.4)	.08
Lifetime smoker, %	14.7	16.1	18.1	24.0	.01
Alcohol misuse, %	10.8	11.0	13.6	17.1	.08
Physically inactive, %	16.2	20.7	26.8	29.8	<.001
Diet (ECI score), mean (SD)^d^	8.61 (1.41)	8.54 (1.44)	8.45 (1.40)	8.37 (1.57)	.04
Antidepressant use, %	4.5	12.0	22.9	37.8	<.001
Anxiolytic use, %	4.2	9.4	19.0	32.5	<.001
Married or cohabiting, %	81.5	77.8	68.4	71.3	<.001
Low social support, %	15.0	19.2	25.5	35.7	<.001
≥10 Stressful life events, %	6.4	12.3	25.4	34.9	<.001
Parental divorce, %	4.6	5.2	4.4	15.0	.01
Parental abuse, %	4.5	6.8	9.4	18.4	<.001
Among the worst childhood cleanliness, %	2.0	2.8	5.1	3.8	.02
Lowest childhood social class (V), %^b^	5.4	7.2	7.4	4.4	.63
Severe adolescent externalization, %	5.7	6.2	7.7	13.3	.08
≥10 wk Childhood sickness absence, %	9.4	12.4	13.2	17.2	.004

Abbreviations: BMI, body mass index (calculated as weight in kilograms divided by height in meters squared); ECI, Eating Choices Index; FEV_1_, forced expiratory volume in 1 second.

^a^ Wald test/joint Wald test *P* value using ordinal logistic regression (unadjusted).

^b^ Unskilled manual occupations.

^c^ Health conditions (as phrased in the questionnaire) included bronchitis, blood pressure problems, heart trouble, cancer, stroke, and diabetes.

^d^ Eating Choices Index scoring ranges from 4 to 20, with higher scores representing healthier choices, based on breakfast consumption, fruit portions, type of milk, and type of bread consumed.

The Figure shows unadjusted survival curves for lifetime case-level affective symptoms. Study members who experienced case-level affective symptoms 3 to 4 times had the lowest survival probability, and those who never experienced case-level symptoms had the highest survival probability.

**Figure.**
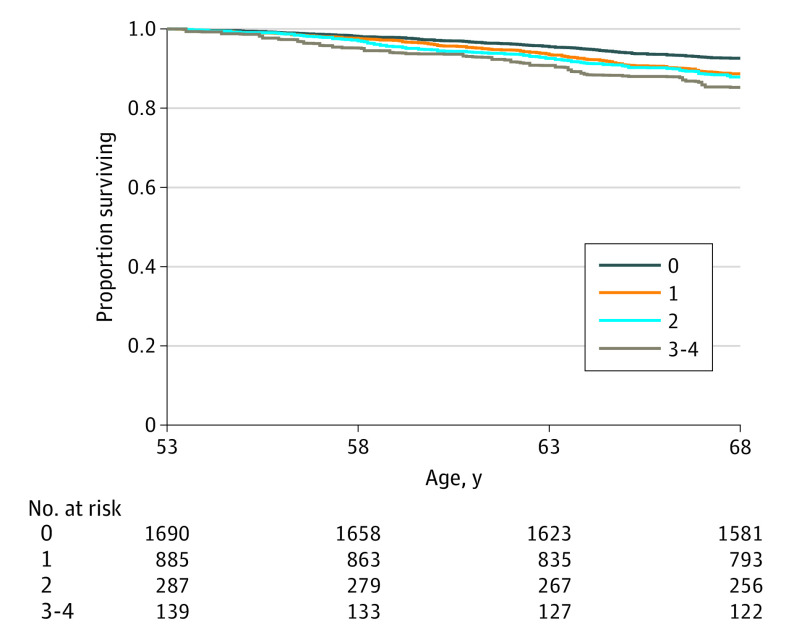
Unadjusted Survival Curves for All-Cause Mortality by Number of Times Case-Level Affective Symptoms Experienced (n = 3001) Number at risk was averaged across 15 imputations and rounded to the nearest whole number.

Table 2 reports that, after adjustment for sex, participants who experienced case-level symptoms 1, 2, and 3 to 4 times had an increased rate of mortality compared with those who never experienced case-level symptoms (76%; aHR, 1.76; 95% CI, 1.29-2.38; aHR, 1.87; 95% CI, 1.18-2.97; and 134%; aHR, 2.34; 95% CI, 1.36-4.04, respectively), and that mortality rates increased with affective case accumulation. After full adjustment, associations between mortality and those who experienced case-level symptoms 2 times (aHR, 1.16; 95% CI, 0.72-1.89) and 3 to 4 times (aHR, 1.18; 95% CI, 0.62-2.25) were largely attenuated. In contrast, the association between those who had case-level symptoms at a single time point and mortality was only partially explained (aHR, 1.46; 95% CI, 1.06-2.02).

**Table 2.** Sex-Adjusted and Fully Adjusted Hazard Ratios for Affective Case Accumulation and All-Cause Mortality^a^

No. of affective cases	Hazard ratio (95% CI)			
	Sex adjusted	Sex-adjusted plus excluded external causes of death^b^	Sex-adjusted plus excluded schizophrenia cases^c^	Fully adjusted^d^
0	1 [Reference]	1 [Reference]	1 [Reference]	1 [Reference]
1	1.76 (1.29-2.38)	1.73 (1.26-2.39)	1.75 (1.29-2.38)	1.46 (1.06-2.02)
2	1.87 (1.18-2.97)	1.87 (1.17-2.97)	1.74 (1.08-2.81)	1.16 (0.72-1.89)
3-4	2.34 (1.36-4.04)	2.33 (1.32-4.10)	2.30 (1.31-4.03)	1.18 (0.62-2.25)

^a^ Based on 15 imputations and 235 deaths (n = 3001).

^b^ Total of 2981 participants, 215 deaths.

^c^ Total of 2984 participants, 231 deaths.

^d^ Adjusted for all covariates listed in Table 1.

Table 3 summarizes how sex-adjusted associations between affective case accumulation and mortality were attenuated by each covariate separately. eTable 2 in the Supplement provides model details. The association between those who had case-level symptoms 3 to 4 times and mortality was most strongly attenuated by the number of health conditions (32.1%), followed by anxiolytic use (28.4%), lung function (24.6%), smoking (24.6%), physical activity (23.9%), antidepressant use (20.1%), diet (16.4%), pulse rate (12.7%), and adult social class (11.2%). An almost identical pattern of attenuation was noted for those who had case-level symptoms twice. In contrast, the association between those who had case-level symptoms once and mortality was attenuated by only 3 variables: number of health conditions (13.2%), lung function (13.2%), and physical activity (11.8%). Many covariates earlier in the life course had little or no explanatory effect.

**Table 3.** Percentage Attenuation of Sex-Adjusted Associations Between Affective Case Accumulation and Mortality by Individual Covariates, Ordered by Total Percentage Attenuation

Covariate	% Attenuation by No. of times affective symptoms were at case level^a^		
	1	2	3-4
Health conditions	13.2	25.3	32.1
Lung function	13.2	28.7	24.6
Physical activity	11.8	25.3	23.9
Anxiolytic use	7.9	18.4	28.4
Smoking	5.3	17.2	24.6
Antidepressant use	6.6	14.9	20.1
Diet	6.6	12.6	16.4
Adult social class	7.9	11.5	11.2
Marital status	6.6	11.5	7.5
Pulse rate	6.6	4.6	12.7
Childhood sickness absence	5.3	4.6	7.5
Educational level	3.9	2.3	6.7
Social support	2.6	3.4	6.0
Childhood cleanliness	2.6	5.7	3.0
Problem drinking	1.3	3.4	5.2
Adolescent externalizing	0	2.3	6.7
Body mass index	1.3	3.4	3.7
Parental abuse	1.3	1.1	3.7
Parental divorce	0	−1.1	4.5
Childhood social class	5.3	0	−3.0
Stressful life events	0	0	0.7
Systolic blood pressure	−1.3	−1.1	−5.2
Fully adjusted (all covariates)	39.5	81.6	86.6

^a^ Percentage attenuation of each covariate was entered separately (ie, not simultaneously) into the sex-adjusted model; since individual covariates are not independent of one another, these figures provide only an approximate indication of the importance of each variable.

Table 4 reports that, after adjustment for sex, case-level affective symptoms in adolescence only were associated with increased mortality compared with those who never had case-level symptoms, which remained after full adjustment (aHR, 1.73; 95% CI, 1.10-2.72). Covariates most strongly attenuating this association were lung function (19.4%), physical activity (19.4%), adult social class (11.2%), pulse rate (11.2%), marital status (11.2%), and childhood sickness–associated absence from school (10.2%). In contrast to all other types of affective case history, self-reported health conditions, anxiolytic use, and antidepressant use had little or no explanatory role (<6% attenuation), whereas smoking appeared to negatively confound the association (−23.5%); eTables 3 and 4 in the Supplement provide model details.

**Table 4.** Sex-Adjusted Hazard Ratios for Affective Case History and All-Cause Mortality by Follow-up Time, Based on 15 Imputations

Variable	Hazard ratio (95% CI)			
	Sex adjusted			Fully adjusted^a^
	15-y Follow-up (n = 3001)	≤4 y of Follow-up (n = 3001)	>4 y of Follow-up (n = 2947)	15-y Follow-up (n = 3001)
Deaths, No.	235	42	193	235
Affective case history^b^				
Never	1 [Reference]	1 [Reference]	1 [Reference]	1 [Reference]
Adolescent only	1.98 (1.27-3.07)	1.17 (0.32-4.29)	2.15 (1.33-3.46)	1.73 (1.10-2.72)
Late onset	1.67 (0.96-2.89)	3.49 (1.27-9.60)	1.26 (0.67-2.37)	1.32 (0.74-2.36)
Intermittent	1.74 (1.26-2.40)	1.24 (0.62-2.94)	1.85 (1.31-2.62)	1.27 (0.89-1.81)
Chronic	2.34 (1.36-4.03)	3.26 (1.10-9.62)	2.11 (1.13-3.97)	1.17 (0.61-2.23)

^a^ Adjusted for all covariates listed in Table 1.

^b^ Never indicates 0 times experienced case-level affective symptoms (reference category); adolescent only, case-level symptoms at ages 13 to 15 years only; late-onset, case-level symptoms at age 53 years only; intermittent, all age groups with case-level symptoms 1 to 2 times; and chronic, case-level symptoms 3 to 4 times.

The Kaplan-Meier graph for affective case history shows that those with late-onset case-level affective symptoms had the lowest survival probability in the first 4 years of follow-up (eFigure in the Supplement). Exploratory analyses suggested that, after adjustment for sex, there was an association between those who had late-onset case-level symptoms and mortality in the first 4 years of follow-up (aHR; 3.49; 95% CI, 1.27-9.60), which was equivalent to that for those who experienced chronic case-level symptoms (aHR, 3.26; 95% CI, 1.10-9.62) but not in later years (aHR, 1.26; 95% CI, 0.67-2.37) (Table 4).

The association between intermittent case-level affective symptoms and mortality (Table 4) was largely attenuated after full adjustment (aHR, 1.27; 0.89-1.81). This attenuation followed a similar pattern noted in participants who experienced case-level symptoms 2 and 3 to 4 times.

Sensitivity analyses (Table 2) showed that the sex-adjusted HR for those who had case-level symptoms twice was slightly attenuated after excluding individuals with schizophrenia (n = 17) (aHR, 1.74; 95% CI, 1.08-2.81); however, all other associations were unaffected. The exclusion of external causes of death (n = 20) did not alter associations.

## Discussion

In a large, British population-based cohort, we found accumulation over the life course where mortality increased with the frequency of case-level affective symptoms (ie, suggestive of a clinical diagnosis): after adjustment for sex, those with case-level affective symptoms 1, 2, and 3 to 4 times had 76%, 87%, and 134% higher rates, respectively, of premature mortality compared with those who never had case-level symptoms over a 15-year follow-up. After full adjustment for covariates, associations between mortality and those who experienced case-level affective symptoms 2 and 3 to 4 times were largely explained; however, the association for participants who had case-level symptoms at a single time point remained. Among the latter group, differential associations were observed. Case-level symptoms in adolescence only (ages 13-15 years) were associated with a 94% increased rate of mortality compared with the rate among those who never had case-level symptoms, which was only partially explained by covariates. In contrast, late-onset case-level symptoms (age 53 years only) demonstrated an association with mortality only in the first 4 years of follow-up. Most associations were explained predominantly by self-reported health conditions, physical activity, lung function, smoking, and psychotropic medication use.

These findings are consistent with previous studies showing a dose-response association between affective symptom accumulation and rates of mortality^4,5^; however, all of these studies were conducted in samples of older persons over a maximum 7-year follow-up.

We found that the association between those who had case-level affective symptoms multiple times (2 and 3-4) and mortality was most strongly attenuated by adult health indicators and behaviors: self-reported health conditions, lung function, physical activity, anxiolytic use, smoking, antidepressant use, diet, pulse rate, and adult social class. In contrast, the association between mortality and case-level symptoms at a single time point was only partially attenuated and by just 3 variables: number of health conditions, lung function, and physical activity. This finding suggests that there could be additional pathways to mortality for those who have case-level symptoms multiple times; for example, the role of behavioral and socioeconomic factors may increase with greater exposure to affective problems. These results are largely consistent with previous studies that found attenuating effects of physical health conditions or illness, physical activity, and smoking.^3,5,25,26^

The association between case-level affective symptoms in adolescence only and mortality was largely unexplained following adjustment for covariates and demonstrated a different pattern of attenuation compared with other affective symptom profiles, suggesting a unique explanatory pathway. We found that late onset of case-level affective symptoms (age 53 years only) showed a strong association with mortality in the first 4 years of follow-up, but not in later years. This finding is consistent with other studies in which recent or new-onset symptoms were associated with mortality,^25,27^ cancer incidence,^28^ and cardiovascular events.^25^

Poor physical health may confound associations between affective symptoms and mortality, particularly in later life, for example, through psychological or inflammatory pathways.^29^ Conversely, affective problems originating earlier in the life course may be associated with detrimental health behaviors (eg, smoking and low levels of physical activity) and subsequent poor health and mortality.^30^ This possibility suggests that health behaviors and poor physical health may mediate the association between chronic affective problems and mortality, but confound associations with regard to adult-onset symptoms; however, it is difficult to infer potential causal pathways in this analysis, as most covariates have a bidirectional association with affective problems.

Several covariates had no notable explanatory role, including parental abuse and neglect. Consistent with these findings, Kelly-Irving et al^31^ found that the association between adverse childhood experiences and mortality was not explained by affective symptoms, suggesting that childhood adversity and affective problems have different pathways to mortality. Also consistent with our findings, population-based studies examining the association between depressive symptoms and mortality have shown a negligible explanatory role for body mass index,^3^ educational level,^3,30^ social support, and stressful life events,^30^ with a moderate role for alcohol abstention (as opposed to misuse).^5^ Adjustment for systolic blood pressure appeared to slightly strengthen associations between affective case accumulation and mortality, which can be attributed to the inverse association between systolic blood pressure–associated affective problems.^32,33^ It is also likely, however, that the attenuating outcome of each variable is modified by cause of death. For example, systolic blood pressure and body mass index might play a stronger role with respect to cardiovascular mortality; this possibility could not be examined in the present study owing to low power.

A primary concern directed at previous studies examining affective symptoms and mortality is a general failure to account for confounding by coexisting mental health problems.^1^ Schizophrenia and adolescent externalizing behavior have demonstrated associations with premature mortality in the NSHD^34^ and elsewhere.^35,36^ However, our results were not altered by adjusting for externalizing behaviors or excluding cases of schizophrenia (n = 25), suggesting that affective problems are associated with mortality rates independent of other mental disorders. Similarly, sensitivity analyses showed that excluding external causes of death had a negligible association with HRs, indicating that individuals who experienced affective problems were no more likely to die by suicide or of unintentional or violent death compared with natural causes; these findings are consistent with previous research.^1,3,25^

### Strengths and Limitations

This study has both strengths and limitations. Strengths of the study include an unusually long follow-up period and prospective data obtained on an extensive range of potential explanatory factors. Mortality data were obtained for nearly all study members using registry linkage data, with missing data addressed using multiple imputation. As with most long-running studies, greater attrition occurred among those with adult social disadvantage and poorer health,^7^ including affective problems, childhood adversity, adolescent externalizing problems, social classes in which individuals had primarily unskilled manual occupations, and men, which could lead to biased estimates, if anything, underestimation of effect sizes. The NSHD data are unique in having repeated measures of affective symptoms from the early 1950s and spanning 4 decades. Each measure is different, yet there is evidence that they capture the same underlying construct: all 4 measures demonstrate a single-factor solution,^9,13,37^ the Present State Examination is highly correlated with the 28-item General Health Questionnaire,^38^ and adolescent affective symptoms in the NSHD are associated with adult psychiatric outcomes at ages 36, 43, and 53 years.^39^ We also provided evidence of dose-response associations between affective case accumulation and many covariates, including psychotropic medication, supporting the validity of this measure.

Conversely, each measure of affective symptoms captured a relatively small window of time, which means that findings for adolescent-only and late-onset cases should be interpreted with caution. Moreover, in contrast to other measures, adolescent-only affective symptoms were rated by a teacher, which could lead to misclassification and potential bias if participants had emotional problems that were unrecognized by teachers; however, teacher ratings have been shown to equally estimate psychiatric disorders compared with self-report in other measures of adolescent emotional problems, such as, the Strength and Difficulties Questionnaire.^40^ Our results are specific to mortality in later life, as causes of death are considerably different earlier in the life course. The generalizability of the results may also be constrained by the NSHD being an all-white cohort. There have also been improvements in mental health awareness and treatment since the 1960s, although access to mental health services remains limited in England.^41^

## Conclusions

We have shown data that suggest accumulation, where increased frequency of case-level affective symptoms are associated with increased mortality. We also found that most associations between lifetime case-level affective symptoms and mortality were primarily explained by self-reported health conditions, lung function, smoking, physical activity, and psychotropic medication use, but that explanatory pathways were likely dependent on both the duration and timing of symptoms. These findings suggest that policies to reduce the interaction between mental and physical health should be aimed predominately at adults as opposed to earlier in the life course. This study also highlights the importance of affective symptom history and may help direct future work in elucidating specific causal pathways and points of intervention.

## Notes

Supplement. **eTable 1.** Descriptive Characteristics of the Observed and Imputed Data **eTable 2.** Multivariable Adjusted Hazard Ratios (95% Confidence Intervals) for the Association Between Affective Case Accumulation and All-Cause Mortality **eTable 3.** Multivariable Adjusted Hazard Ratios (95% Confidence Intervals) for the Association Between Affective Case History and All-Cause Mortality **eTable 4.** Percentage Attenuation of the Sex Adjusted Association Between Affective Case History and Mortality, by Individual Covariates (Ordered by Total Attenuation) **eFigure.** Unadjusted Survival Curves for All-Cause Mortality by Affective Case History
