# Supplementary material for: Association Between Lifetime Affective Symptoms and Premature Mortality
Source: JAMA Psychiatry. 2020 Apr 8;77(8):1–8. doi: 10.1001/jamapsychiatry.2020.0316 (PMC7142795; doi:10.1001/jamapsychiatry.2020.0316)
Supplement: Supplement. — eTable 1. Descriptive Characteristics of the Observed and Imputed Data eTable 2. Multivariable Adjusted Hazard Ratios (95% Confidence Intervals) for the Association Between Affective Case Accumulation and All-Cause Mortality eTable 3. Multivariable Adjusted Hazard Ratios (95% Confidence Intervals) for the Association Between Affective Case History and All-Cause Mortality eTable 4. Percentage Attenuation of the Sex Adjusted Association Between Affective Case History and Mortality, by Individual Covariates (Ordered by Total Attenuation) eFigure. Unadjusted Survival Curves for All-Cause Mortality by Affective Case History [file jamapsychiatry-77-806-s001.pdf]

# Supplementary Online Content

Archer G, Kuh D, Hotopf M, Stafford M, Richards M. Association between lifetime affective symptoms and premature mortality. *JAMA Psychiatry*. Published online April 8, 2020. doi:10.1001/jamapsychiatry.2020.0316

**eTable 1.** Descriptive Characteristics of the Observed and Imputed Data

**eTable 2.** Multivariable Adjusted Hazard Ratios (95% Confidence Intervals) for the Association Between Affective Case Accumulation and All-Cause Mortality

**eTable 3.** Multivariable Adjusted Hazard Ratios (95% Confidence Intervals) for the Association Between Affective Case History and All-Cause Mortality

**eTable 4.** Percentage Attenuation of the Sex Adjusted Association Between Affective Case History and Mortality, by Individual Covariates (Ordered by Total Attenuation)

**eFigure.** Unadjusted Survival Curves for All-Cause Mortality by Affective Case History

This supplementary material has been provided by the authors to give readers additional information about their work.

© 2020 Archer G et al. *JAMA Psychiatry.*

## eTable 1. Descriptive characteristics of the observed and imputed data

| | Original (n=2066) | Imputed (n=3001) |
|---|---|---|
| | No. (%) | % |
| **Affective symptom case**[a] | | |
| 0 | 1195 (57.8) | 56.4 |
| 1 | 601 (29.1) | 29.4 |
| 2 | 182 (8.8) | 9.7 |
| 3-4 | 88 (4.3) | 4.5 |
| Missing | 935 | |
| **Affective symptom history**[ab] | | |
| Adolescent only | 165 (9.3) | 8.7 |
| Intermittent | 446 (23.8) | 23.0 |
| Adult-onset | 165 (7.2) | 8.3 |
| Missing | 935 | |
| **Sex** | | |
| Male | 1014 (49.7) | 49.7 |
| Female | 1052 (50.3) | 50.3 |
| Missing | 0 | |
| **Social class (adult)** | | |
| Professional (I) | 212 (10.6) | 10.2 |
| Intermediate (II) | 783 (39.6) | 38.8 |
| Skilled, non-manual (III-NM) | 263 (12.2) | 12.2 |
| Skilled, manual (III-M) | 507 (25.0) | 25.6 |
| Partly skilled (IV) | 180 (9.1) | 9.5 |
| Unskilled (V) | 68 (3.5) | 3.7 |
| Missing | 53 | |
| **Education** | | |
| None | 736 (36.6) | 36.6 |
| O-level or equivalent | 577 (28.4) | 28.4 |
| A-level or equivalent | 513 (25.5) | 25.5 |
| Higher education | 194 (9.6) | 9.5 |
| Missing | 43 | |
| **Systolic blood pressure** (mmHg) | | |
| Mean (SD) | 137.6 (20.9) | 137.8 (21.02) |
| Missing | 36 | |
| **Lung function** ($FEV_1$) | | |
| Mean (SD) | 2.70 (0.71) | 2.70 (0.72) |
| Missing | 51 | |
| **Pulse rate** (bpm) | | |
| Mean (SD) | 68.1 (11.53) | 68.3 (11.60) |
| Missing | 49 | |
| **Body mass index** (kg/m2) | | |
| Mean (SD) | 27.5 (4.78) | 27.5 (4.79) |
| Missing | 21 | |

© 2020 Archer G et al. *JAMA Psychiatry.*

| (continued.) | Original (n=2066) | Imputed (n=3001) |
|---|---|---|
| | **No. (%)** | **%** |
| **Health conditions** | | |
| 0 | 1023 (48.8) | 48.2 |
| 1 | 603 (30.3) | 30.3 |
| 2 | 275 (13.7) | 13.9 |
| 3+ | 141 (7.3) | 7.6 |
| Missing | 24 | |
| **Diet (ECI)[a]** | | |
| Mean (SD) | 8.81 (1.37) | 8.56 (1.43) |
| Missing | 947 | |
| **Smoking history** | | |
| Never smoked | 587 (28.5) | 28.4 |
| Predominantly non-smoker | 717 (34.1) | 34.1 |
| Predominantly smoker | 440 (21.6) | 21.6 |
| Lifetime smoker | 298 (15.8) | 15.8 |
| Missing | 24 | |
| **Problem drinking** | | |
| No | 1824 (88.6) | 88.6 |
| Yes | 242 (11.4) | 11.4 |
| Missing | 0 | |
| **Physical activity[a]** | | |
| Inactive | 376 (18.6) | 19.2 |
| Moderately active | 984 (46.8) | 46.7 |
| Most active | 702 (34.6) | 34.1 |
| Missing | 4 | |
| **Antidepressant use** | | |
| No | 1743 (90.9) | 90 |
| Yes | 152 (9.2) | 10 |
| Missing | 171 | |
| **Anxiolytic use** | | |
| No | 1782 (92.8) | 91.5 |
| Yes | 106 (7.2) | 8.5 |
| Missing | 178 | |
| **Marital status** | | |
| Married or cohabiting | 1653 (79.2) | 78.7 |
| Single | 100 (5.0) | 5.4 |
| Divorced, widowed, separated | 312 (15.7) | 15.9 |
| Missing | 1 | |
| **Social support (friends)[a]** | | |
| Always | 1703 (82.3) | 81.8 |
| Often/Sometimes/ Never | 359 (17.7) | 18.2 |
| Missing | 4 | |
| **Stressful life events** | | |
| 0-5 | 1087 (55.8) | 55.5 |

© 2020 Archer G et al. *JAMA Psychiatry.*

| (continued.) | Original (n=2066) | Imputed (n=3001) |
|---|---|---|
| | **No. (%)** | **%** |
| **Stressful life events** | | |
| 6-10 | 647 (33.4) | 33.3 |
| 10+ | 203 (10.8) | 11.3 |
| Missing | 129 | |
| **Social class (childhood)** | | |
| Professional (I) | 132 (6.2) | 6.2 |
| Intermediate (II) | 391 (19.4) | 19.5 |
| Skilled, nonmanual (III-NM) | 323 (16.3) | 16.4 |
| Skilled, manual (III-M) | 668 (33.0) | 32.9 |
| Partly skilled (IV) | 401 (19.0) | 18.8 |
| Unskilled (V) | 124 (6.1) | 6.1 |
| Missing | 27 | |
| **Externalising (age 13-15)** | | |
| None/mild | 1620 (76.9) | 76.6 |
| Moderate | 321 (16.6) | 17 |
| Severe | 125 (6.6) | 6.4 |
| Missing | 0 | |
| **Childhood sickness absence (weeks)** | | |
| 0-4 | 894 (52.9) | 53.1 |
| 4-10 | 625 (36.3) | 36 |
| 10+ | 192 (10.9) | 11 |
| Missing | 355 | |
| **Cleanliness of child** | | |
| Amongst the best/ average | 1878 (97.7) | 97.4 |
| Amongst the worst | 47 (2.3) | 2.6 |
| Missing | 141 | |
| **Parental abuse** | | |
| No | 1837 (94.1) | 93.7 |
| Yes | 106 (5.9) | 6.3 |
| Missing | 123 | |
| **Parental divorce** | | |
| No | 1972 (94.7) | 94.7 |
| Yes | 94 (5.3) | 5.3 |
| Missing | 0 | |

a: Variables derived following the imputation process using measures at multiple time-points
b: Adolescent-only = case at age 13-15 only; late-onset = case at age 53 only; intermittent = all others who were a case 1-2 times

© 2020 Archer G et al. *JAMA Psychiatry.*

**eTable 2. Multivariable adjusted hazard ratios (95% confidence intervals) for the association between affective case accumulation and all-cause mortality; based on 15 imputations, 235 deaths and a 15-year follow-up (n = 3001)**

| | **Number of times an affective case** | | | |
|---|---|---|---|---|
| | **None** | **1** | **2** | **3-4** |
| Sex adjusted | 1 (ref) | 1.76 (1.29, 2.38) | 1.87 (1.18, 2.97) | 2.34 (1.36, 4.04) |
| Sex + adult social class | 1 | 1.70 (1.25, 2.31) | 1.77 (1.12, 2.79) | 2.19 (1.26, 3.78) |
| Sex + education | 1 | 1.73 (1.27, 2.34) | 1.85 (1.17, 2.94) | 2.25 (1.31, 3.89) |
| Sex + systolic blood pressure | 1 | 1.77 (1.31, 2.41) | 1.88 (1.19, 2.98) | 2.41 (1.40, 4.14) |
| Sex + lung function | 1 | 1.66 (1.22, 2.27) | 1.62 (1.02, 2.56) | 2.01 (1.16, 3.49) |
| Sex + pulse rate | 1 | 1.71 (1.26, 2.32) | 1.83 (1.16, 2.89) | 2.17 (1.26, 3.75) |
| Sex + body mass index | 1 | 1.75 (1.29, 2.37) | 1.84 (1.16, 2.92) | 2.29 (1.33, 3.96) |
| Sex + health conditions | 1 | 1.66 (1.22, 2.25) | 1.65 (1.04, 2.60) | 1.91 (1.10, 3.34) |
| Sex + diet | 1 | 1.71 (1.26, 2.31) | 1.76 (1.12, 2.79) | 2.12 (1.21, 3.70) |
| Sex + smoking | 1 | 1.72 (1.26, 2.34) | 1.72 (1.09, 2.73) | 2.01 (1.16, 3.48) |
| Sex + problem drinking | 1 | 1.75 (1.29, 2.38) | 1.84 (1.16, 2.91) | 2.27 (1.32, 3.92) |
| Sex + physical activity | 1 | 1.67 (1.23, 2.27) | 1.65 (1.04, 2.62) | 2.02 (1.17, 3.51) |
| Sex + anxiolytic use | 1 | 1.70 (1.25, 2.31) | 1.71 (1.07, 2.72) | 1.96 (1.11, 3.46) |
| Sex + antidepressant use | 1 | 1.71 (1.26, 2.31) | 1.74 (1.10, 2.77) | 2.07 (1.16, 3.69) |
| Sex + marital status | 1 | 1.71 (1.25, 2.33) | 1.77 (1.12, 2.81) | 2.24 (1.30, 3.86) |
| Sex + social support | 1 | 1.74 (1.28, 2.37) | 1.84 (1.16, 2.92) | 2.26 (1.30, 3.92) |
| Sex + stressful life events | 1 | 1.76 (1.29, 2.40) | 1.87 (1.16, 3.00) | 2.33 (1.33, 4.07) |
| Sex + childhood cleanliness | 1 | 1.74 (1.29, 2.37) | 1.82 (1.15, 2.88) | 2.30 (1.33, 3.97) |
| Sex + parental abuse | 1 | 1.75 (1.29, 2.38) | 1.86 (1.18, 2.95) | 2.29 (1.32, 3.96) |
| Sex + parental divorce | 1 | 1.76 (1.29, 2.38) | 1.88 (1.19, 2.97) | 2.28 (1.32, 2.95) |
| Sex + childhood social class | 1 | 1.72 (1.27, 2.33) | 1.87 (1.18, 2.97) | 2.38 (1.38, 4.10) |
| Sex + adolescent externalising | 1 | 1.76 (1.30, 2.39) | 1.85 (1.17, 2.93) | 2.25 (1.30, 3.90) |
| Sex + childhood sickness absence | 1 | 1.72 (1.27, 2.34) | 1.83 (1.15, 2.89) | 2.24 (1.30, 3.88) |
| Fully adjusted (all covariates) | 1 | 1.46 (1.06, 2.02) | 1.16 (0.72, 1.89) | 1.18 (0.62, 2.25) |

© 2020 Archer G et al. *JAMA Psychiatry.*

**eTable 3. Multivariable adjusted hazard ratios (95% confidence intervals) for the association between affective case history and all-cause mortality; based on 15 imputations, 235 deaths and a 15-year follow-up (n = 3001)**

| | Affective case history[a] | | | | |
|---|---|---|---|---|---|
| | **Never** | **Adolescent only** | **Late-onset** | **Intermittent** | **Chronic** |
| Sex adjusted | 1 (ref) | 1.98 (1.27-3.07) | 1.67 (0.96-2.89) | 1.74 (1.26-2.40) | 2.34 (1.36-4.03) |
| Sex+ adult social class | 1 | 1.87 (1.20, 2.90) | 1.61 (0.93, 2.79) | 1.68 (1.22, 2.32) | 2.18 (1.26, 3.78) |
| Sex + education | 1 | 1.90 (1.23, 2.96) | 1.64 (0.95, 2.85) | 1.73 (1.25, 2.38) | 2.25 (1.30, 3.89) |
| Sex + systolic blood pressure | 1 | 1.98 (1.28, 3.07) | 1.70 (0.98, 2.95) | 1.76 (1.27, 2.42) | 2.40 (1.39, 4.14) |
| Sex + lung function | 1 | 1.79 (1.15, 2.78) | 1.65 (0.95, 2.88) | 1.60 (1.15, 2.21) | 2.01 (1.15, 3.48) |
| Sex + pulse rate | 1 | 1.87 (1.21, 2.91) | 1.64 (0.95, 2.84) | 1.71 (1.24, 2.37) | 2.17 (1.26, 3.74) |
| Sex + body mass index | 1 | 1.99 (1.29, 3.09) | 1.64 (0.95, 2.85) | 1.72 (1.25, 2.37) | 2.29 (1.32, 3.95) |
| Sex + health conditions | 1 | 1.93 (1.24, 3.00) | 1.54 (0.89, 2.67) | 1.58 (1.14, 2.18) | 1.90 (1.09, 3.32) |
| Sex + diet | 1 | 1.90 (1.23, 2.94) | 1.67 (0.96, 2.91) | 1.65 (1.20, 2.28) | 2.11 (1.21, 3.69) |
| Sex + smoking | 1 | 2.21 (1.42, 3.45) | 1.57 (0.90, 2.74) | 1.59 (1.15, 2.20) | 1.99 (1.15, 3.45) |
| Sex + problem drinking | 1 | 2.02 (1.30, 3.13) | 1.67 (0.96, 2.89) | 1.70 (1.23, 2.35) | 2.26 (1.31, 3.91) |
| Sex + physical activity | 1 | 1.79 (1.15, 2.78) | 1.60 (0.92, 2.78) | 1.63 (1.18, 2.26) | 2.02 (1.17, 3.51) |
| Sex + anxiolytic use | 1 | 1.98 (1.27, 3.07) | 1.63 (0.94, 2.83) | 1.60 (1.15, 2.23) | 1.93 (1.09, 3.43) |
| Sex + antidepressant use | 1 | 1.97 (1.27, 3.04) | 1.60 (0.93, 2.77) | 1.64 (1.18, 2.28) | 2.05 (1.15, 3.66) |
| Sex + marital status | 1 | 1.87 (1.20, 2.92) | 1.65 (0.95, 2.86) | 1.68 (1.22, 2.33) | 2.24 (1.30, 3.86) |
| Sex + social support | 1 | 1.96 (1.26, 3.04) | 1.65 (0.95, 2.86) | 1.72 (1.24, 2.37) | 2.25 (1.30, 3.91) |
| Sex + stressful life events | 1 | 1.97 (1.27, 3.06) | 1.67 (0.96, 2.91) | 1.73 (1.24, 2.42) | 2.30 (1.31, 4.02) |
| Sex + childhood cleanliness | 1 | 1.96 (1.26, 3.04) | 1.66 (0.96, 2.87) | 1.71 (1.24, 2.36) | 2.30 (1.33, 3.97) |
| Sex + parental abuse | 1 | 1.97 (1.27, 3.06) | 1.67 (0.96, 2.89) | 1.73 (1.25, 2.39) | 2.28 (1.32, 3.96) |
| Sex + parental divorce | 1 | 1.98 (1.28, 3.07) | 1.66 (0.96, 2.88) | 1.74 (1.26, 2.40) | 2.27 (1.31, 3.94) |
| Sex + childhood social class | 1 | 1.89 (1.22, 2.95) | 1.62 (0.93, 2.81) | 1.73 (1.26, 2.39) | 2.38 (1.38, 4.09) |
| Sex + adolescent externalising | 1 | 2.04 (1.31, 3.16) | 1.66 (0.96, 2.88) | 1.71 (1.24, 2.36) | 2.24 (1.29, 3.89) |
| Sex + childhood sickness absence | 1 | 1.88 (1.21, 2.92) | 1.63 (0.94, 2.83) | 1.73 (1.25, 2.39) | 2.24 (1.29, 3.88) |
| Fully adjusted (all covariates) | 1 | 1.73 (1.10, 2.72) | 1.32 (0.74, 2.36) | 1.27 (0.89, 1.81) | 1.17 (0.61, 2.23) |

**a**: Never = 0 times a case (ref); adolescent-only = case at age 13-15 only; late-onset = case at age 53 only; intermittent = all others who were a case 1-2 times; chronic = 3-4 times a case

© 2020 Archer G et al. *JAMA Psychiatry.*

## eTable 4. Percentage attenuation of the sex adjusted association between affective case history and mortality, by individual covariates (ordered by total attenuation)

| | % attenuation[b] | | | |
|---|---|---|---|---|
| **Covariate** | **Adolescent only** | **Late-onset** | **Intermittent** | **Chronic** |
| Self-reported health conditions | 5.1 | 19.4 | 21.6 | 32.8 |
| Physical activity | 19.4 | 10.4 | 14.9 | 23.9 |
| Lung function | 19.4 | 3.0 | 18.9 | 24.6 |
| Smoking | -23.5 | 14.9 | 20.3 | 26.1 |
| Anxiolytic use | 0.0 | 6.0 | 18.9 | 30.6 |
| Antidepressant use | 1.0 | 10.4 | 13.5 | 21.6 |
| Diet | 8.2 | 0.0 | 12.2 | 17.2 |
| Adult social class | 11.2 | 9.0 | 8.1 | 11.9 |
| Pulse rate | 11.2 | 4.5 | 4.1 | 12.7 |
| Marital status | 11.2 | 3.0 | 8.1 | 7.5 |
| Childhood sickness absence | 10.2 | 6.0 | 1.4 | 7.5 |
| Education | 8.2 | 4.5 | 1.4 | 6.7 |
| Childhood social class | 9.2 | 7.5 | 1.4 | -3.0 |
| Social support (friends) | 2.0 | 3.0 | 2.7 | 6.7 |
| Adolescent externalising | -6.1 | 1.5 | 4.1 | 7.5 |
| Systolic blood pressure | 0.0 | -4.5 | -2.7 | -4.5 |
| Problem drinking | -4.1 | 0.0 | 5.4 | 6.0 |
| Childhood cleanliness | 2.0 | 1.5 | 4.1 | 3.0 |
| Body mass Index | -1.0 | 4.5 | 2.7 | 3.7 |
| Parental abuse | 1.0 | 0.0 | 1.4 | 4.5 |
| Parental divorce | 0.0 | 1.5 | 0.0 | 5.2 |
| Stressful life events | 1.0 | 0.0 | 1.4 | 3.0 |
| Fully adjusted (all covariates) | 23.5 | 53.7 | 62.2 | 85.1 |

**a:** Never = 0 times a case; adolescent-only = case at age 13-15 only; late-onset = case at age 53 only; intermittent = all others who were a case 1-2 times; chronic = 3-4 times a case
**b:** % attenuation of each individual covariate entered separately into the sex-adjusted mode; since individual covariates are not independent of one another, these figures provide only an approximate indication of the importance of each variable

© 2020 Archer G et al. *JAMA Psychiatry.*

**eFigure. Unadjusted survival curves for all-cause mortality by affective case history (n = 3001)**

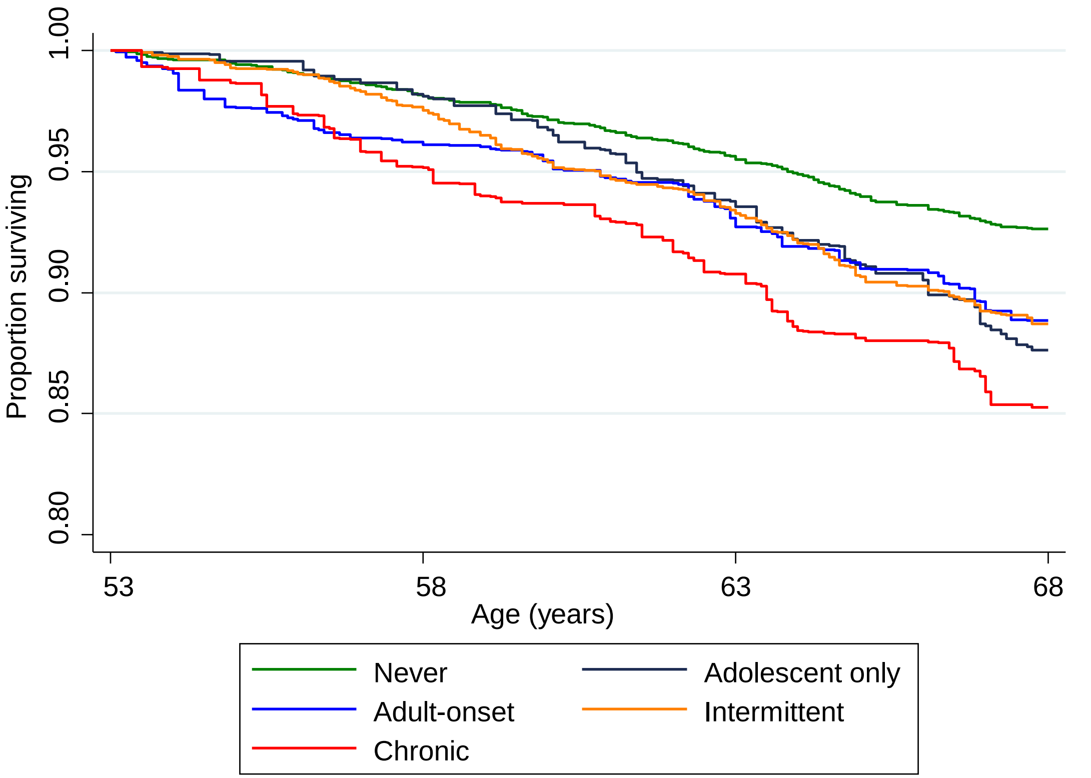


No. at risk*

| | | | | |
|---|---|---|---|---|
| Never | 1690 | 1658 | 1623 | 1581 |
| Adolescent-only | 265 | 260 | 249 | 234 |
| Intermittent | 222 | 212 | 207 | 199 |
| Adult-onset | 685 | 670 | 645 | 617 |
| Chronic | 139 | 133 | 127 | 122 |

*averaged across 15 imputations and rounded to nearest whole number

© 2020 Archer G et al. *JAMA Psychiatry.*
